# Biopsychosocial risk factors for pain in early phases of pediatric cancer treatment

**DOI:** 10.3389/fpsyg.2025.1507560

**Published:** 2025-04-29

**Authors:** Kimberly L. Klages, Ryan N. James, Zeev N. Kain, Sean Phipps, Nicole M. Alberts, Michelle A. Fortier

**Affiliations:** ^1^Division of Behavioral Medicine and Clinical Psychology and the Cancer and Blood Diseases Institute, Cincinnati Children's Hospital Medical Center, Cincinnati, OH, United States; ^2^Department of Psychology and Biobehavioral Sciences, St. Jude Children's Research Hospital Medical Center, Memphis, TN, United States; ^3^Center on Stress and Health, University of California Irvine School of Medicine, Irvine, CA, United States; ^4^Department of Anesthesiology & Perioperative Care, University of California Irvine, Irvine, CA, United States; ^5^Department of Pediatrics, CHOC Children's Hospital, Orange, CA, United States; ^6^Department of Psychology, Concordia University, Montreal, QC, Canada; ^7^Department of Pediatric Psychology, Sue & Bill Gross School of Nursing, University of California Irvine, Irvine, CA, United States

**Keywords:** pediatric cancer, pain, biopsychosocial, pain assessment, pain management

## Abstract

**Introduction:**

Cancer pain remains a significant burden among children with cancer, and many patients experience pain starting around the time of diagnosis and throughout the course of treatment. A biopsychosocial treatment approach has been recommended to improve pain management in this population; however, specific psychosocial factors that contribute to pain in the early phases of pediatric cancer treatment have yet to be identified. The purpose of this study was to explore the biopsychosocial factors associated with pain experiences during the early phases of pediatric cancer treatment, with the goal of identifying children who may be at the highest risk for pain to inform future intervention and prevention efforts.

**Methods:**

Data were collected from 203 children with cancer (*M* = 12.3 years of age, 53.2% male, 41.4% White, 26.6% Latino) and their primary caregiver within the first several weeks of treatment (*M* = 10 weeks). Children completed self-report questionnaires and caregivers completed self- and parent-proxy questionnaires at baseline. Cancer-related data, including diagnosis and date of diagnosis, were abstracted from the child's electronic medical record. Multiple regression analysis was used to examine associations between biopsychosocial risk factors, pain intensity, and pain interference.

**Results:**

Older age, female gender, and elevated depressive symptoms, fatigue, and child self-reported pain catastrophizing were significantly associated with increased pain intensity. Additionally, lower annual income, decreased physical functioning, and greater fatigue, child pain catastrophizing, and parent stress were significantly associated with increased pain interference.

**Discussion:**

Findings underscore the importance of utilizing a comprehensive biopsychosocial approach to pain assessment and management in pediatric oncology. This approach highlights the need for targeted interventions that address not only the physical aspects of pain but also the psychological and social contexts of patients, ensuring a more holistic and effective treatment strategy.

## Introduction

Over 12,000 children and adolescents are diagnosed with cancer each year in the United States (Siegel et al., 2021). Pediatric cancer survival rates have greatly improved with advancements in treatment; however, cancer pain remains a significant burden and has been reported by children and their caregivers as the most distressing symptom of their cancer experience (Collins et al., 2000; Hedstrom et al., 2003; Moody et al., 2006; Pöder et al., 2010; Dupuis et al., 2016; Tutelman et al., 2018; Jibb et al., 2022). Pediatric patients may experience pain starting around the time of diagnosis, as well as throughout the course of treatment (Jibb et al., 2022; Elliott et al., 1991; Forgeron et al., 2006; Levine et al., 2017; Miser et al., 1987; Zernikow et al., 2005). Moreover, unmanaged pain during treatment has been shown to decrease quality of life, coinciding with various adverse outcomes (e.g., sleep problems, stress, depression, development of behavior problems) (Tutelman et al., 2018; Ruccione et al., 2013; Simons et al., 2014). In addition, pediatric cancer patients are at greater risk of being diagnosed with a pain condition and other health-related complications into survivorship (Diller et al., 2009; Huang et al., 2013; Lu et al., 2011; Alberts et al., 2018, 2024).

Pain is a complex experience that involves multiple factors and contributors. Accordingly, the biopsychosocial model describes pain as multidimensional and involving dynamic interactions among biological, psychological, and social factors (Simons et al., 2014; Gatchel et al., 2014). Specific psychosocial factors may serve as risk or resilience factors that influence the probability of developing a chronic pain condition (Meints and Edwards, 2018). For example, psychological factors such as anxiety and depressive symptoms, fatigue, and pain catastrophizing, can increase pain and pain-related distress, whereas engagement in physical activity, lower pain-related disability, and high pain self-efficacy (i.e., engaging in routine activity despite pain) can reduce pain (Meints and Edwards, 2018). Specific biological and social factors, such as older age, female gender, and lower socioeconomic status, have also been identified as risk factors for the development of chronic pain (de Oliveira et al., 2023; Mills et al., 2019; Schmitz et al., 2013; Huguet et al., 2016; Vierhaus et al., 2011). The identification of the biopsychosocial factors associated with pain is a crucial element in optimizing pain outcomes (Kovačević et al., 2024), and, as such, it is recommended that the understanding and treatment of pain include a comprehensive assessment that incorporates a biopsychosocial perspective (Meints and Edwards, 2018; Gatchel et al., 2007; Liossi and Howard, 2016; Bevers et al., 2016).

Literature examining pain in pediatric cancer patients supports embracing a multifaceted, biopsychosocial approach to improve pain management for this population (Fuller et al., 2022; Klages et al., 2025). However, the early weeks of cancer treatment present a unique context in which pain experiences may differ from those observed in pediatric chronic pain populations. Unlike chronic pain conditions that develop over time, pain during early cancer treatment is often acute and may arise from a combination of disease-related factors (e.g., tumor infiltration, inflammation), intensive medical procedures (e.g., surgeries, lumbar punctures, chemotherapy-related mucositis or neuropathy), and significant psychological stress associated with a new cancer diagnosis (Alberts et al., 2018; Coluzzi et al., 2020; Ribeiro et al., 2017). This period is also marked by substantial physiological changes, including immune suppression and metabolic alterations, which may further influence pain sensitivity and processing (Page, 2013; Silva Santos Ribeiro et al., 2022; Lin et al., 2022). Additionally, distress related to repeated painful experiences during treatment may contribute to fear and avoidance behaviors, potentially shaping long-term pain trajectories differently than in youth with non-cancer chronic pain conditions (Fuller et al., 2022; Uhl et al., 2020). Caregivers also experience considerable distress while assisting their child in identifying effective pain management strategies (Twycross et al., 2015; Caes et al., 2014). Given these unique factors, relying solely on findings from the pediatric chronic pain literature may not fully capture the complexities of pain in this specific context. Instead, early, targeted interventions tailored to the distinct biological, psychological, and treatment-related contributors to pain in pediatric oncology are needed to optimize pain control and prevent long-term adverse outcomes (Gatchel et al., 2014; Bevers et al., 2016).

**Figure 1:**
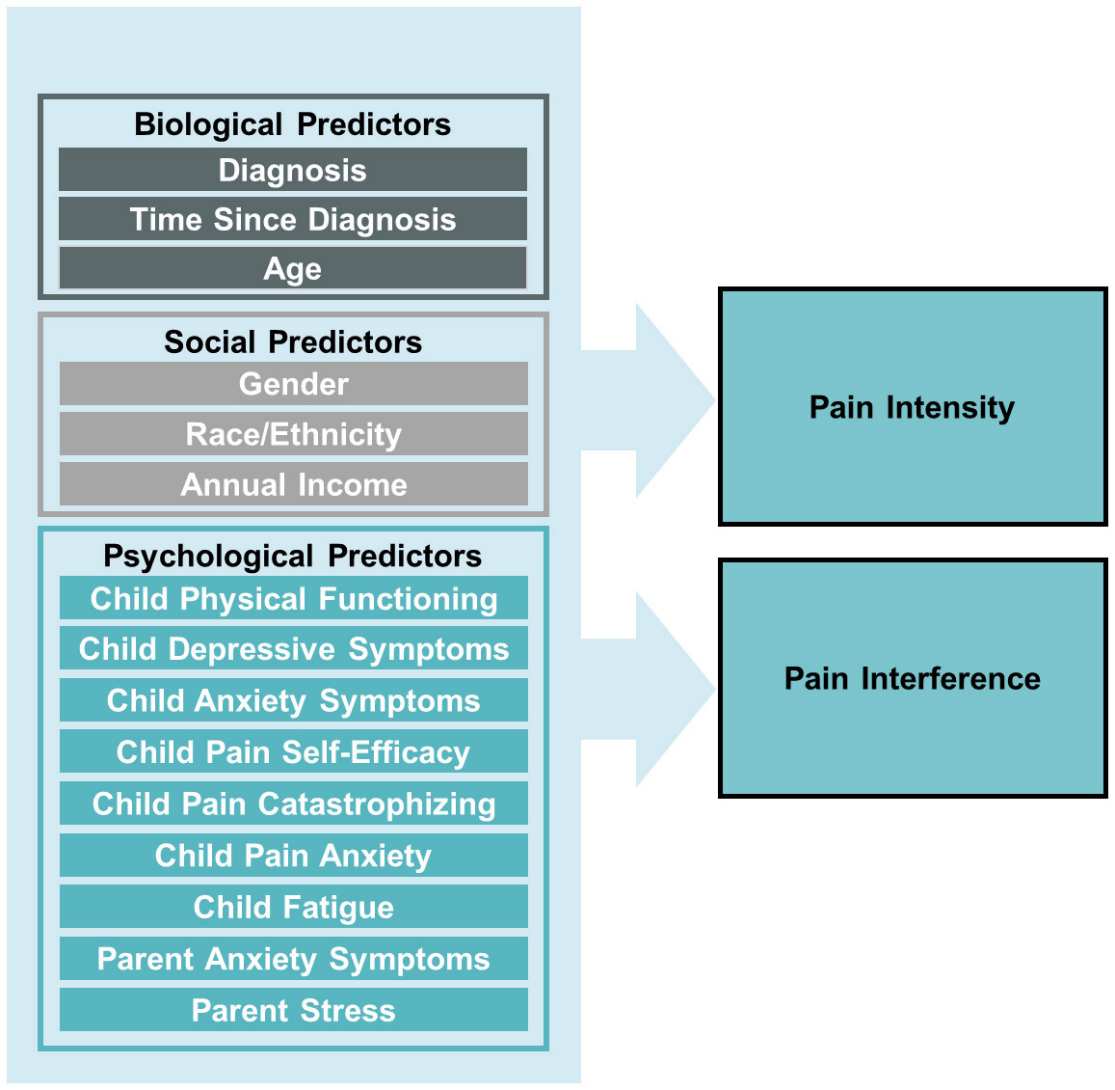
Conceptual model of biopsychosocial predictors of pain intensity and pain interference in children newly diagnosis with cancer.

Despite significant pain experienced by youth with cancer, as well as interest to improve pain-related distress in this population, examination of specific biopsychosocial factors that may contribute to pain in the early phases of pediatric cancer treatment remain understudied. As such, the purpose of the current study was to explore biopsychosocial factors that may be associated with the pain experience of children during early phases of cancer treatment. Specifically, we aimed to identify biopsychosocial factors that may assist in the identification of children early in the cancer journey who may be most at-risk for pain and pain-related disability (see Figure 1 for conceptual model) in order to aid in intervention and ultimately, prevention of cancer-related pain. We hypothesized that older age, female gender, lower annual income, greater psychological distress (e.g., anxiety, depressive symptoms, pain catastrophizing, parental stress), increased fatigue, and lower pain self-efficacy and physical functioning would be associated with higher pain intensity and greater pain-related disability during early cancer treatment.

## Materials and methods

### Participants

This trial was approved by the Institutional Review Boards at both recruitment sites and registered with ClinicalTrials.gov (ClinicalTrials.gov Protocol Record NCT03384134). While the data of this manuscript are from a randomized controlled trial of a mobile health (mHealth) intervention designed to reduce pain and symptoms, the results presented in this manuscript reflect only data collected at recruitment (baseline) only and prior to randomization into the two intervention groups. Eligible patients were recruited from two major pediatric cancer centers in the United States, one in the western region and the other in the mid-southern region. Participants were eligible if they were ages 8-18 years old and: (a) were within 16 weeks of a first time cancer diagnosis, (b) primarily receiving or likely to primarily receive outpatient anti-cancer therapy, (c) were fluent in English, (d) had experienced a pain score of 30 or greater on a visual analog scale (VAS) at recruitment, and (e) had home internet access to use the mHealth intervention. Exclusion criteria included: (a) cognitive impairment of the child (e.g., developmental delay) that would impact the child's ability to use the mHealth program and (b) children whose treatment protocols were largely inpatient. A total of 285 patients meeting the initial eligibility criteria (a–c above) were approached. Of these, 67 reported VAS scores below 30 at recruitment, resulting in a final sample of 203 participants.

### Measures

#### Demographic and baseline data

Parents completed a questionnaire assessing a range of demographic data, including children's age, gender, and race/ethnicity as well as annual household income. Cancer diagnosis and date of diagnosis were extracted from the child's electronic medical record.

#### Pain: visual analog scale (VAS)

Because of the focus on pain reduction in the overall trial, children who reported a 30 or greater on the VAS were eligible for recruitment. The VAS collects pain severity using a 0-100 scale and is a recommended measure of self-report pain in children ages 8 and older and has good psychometric properties (Savedra et al., 1989).

#### Health-related quality of life: pediatric quality of life inventory (PedsQL)

The present study incorporated by the generic and multidimensional fatigue modules of the PedsQL, which are widely used and well-validated measures of health-related quality of life applied to healthy children and adolescents and those with acute and chronic health conditions (Varni et al., 2002; Varni and Limbers, 2009). Children respond to items using a 5-point Likert-type scale that ranges from 0 (*never a problem for me*) to 4 (*almost always a problem for me*). Items are reverse-scored and then transformed to a 0-100 scale with higher scores reflecting better quality of life. Psychometric properties for the PedsQL are excellent (Varni et al., 2002; Varni and Limbers, 2009). Children completed the self-report version of the PedsQL and parents completed the parent proxy report of children's functioning.

#### Pain-related functioning: child activities limitation interview (CALI-21)

The CALI-21 is a validated measure of pain-related functional impairment in school-aged children (Palermo et al., 2008). Children are asked to respond to pain-related limitations in 21 functional activities using a 5-point Likert-type scale where 0 = *not difficult* to 4 = *extremely difficult*. Higher scores reflect greater pain-related functional impairment. The CALI-21 has strong psychometric properties and is a widely used measure in children with chronic pain (Palermo et al., 2008).

#### Anxiety and depressive symptoms: revised child anxiety and depression scale (RCADS)

The RCADS is a well-validated and widely used measure to assess potentially clinical levels of anxiety and depression in youth (Chorpita et al., 2000). Both anxiety and depression are highly associated with chronic pain in children and accordingly, was assessed as a potential correlate of pain in the present study. The RCADS contains 47 items, rated on a 4-point Likert-type scale (0 = *never*, 3 = *always*) and has strong psychometric properties (Chorpita et al., 2005).

#### Pain catastrophizing: pain catastrophizing scale for children (PCS-C)

Given strong associations between children's pain and catastrophizing, which reflects children's worry, focus on negative outcomes, and feelings of inability to cope with pain. Pain catastrophizing has been shown to be associated with higher pain severity in the context of chronic pain and is a predictor of both persistent pain and the transition from acute to chronic pain (Kremer et al., 2013). The PCS-C is a well-validated measure of children's pain catastrophizing that has children respond to items using a 5-point Likert-type scale ranging from 0 (*not at all*) to 4 (*very true*) with higher scores reflecting greater catastrophic thinking (Crombez Bijttebier et al., 2003).

#### Parental stress: perceived stress scale (PSS)

Given increased parental stress as a function of caring for a child with cancer and potential associations with parental stress and children's pain, we assessed parent stress with the PSS, a widely used self-report measure in which individuals report the degree to which parents perceive their lives as stressful within the last month (Cohen et al., 1983). The PSS is a 14-item measure in which parents responded to items using a 5-point Likert-type scale where 0 = *never* and 4 = *very often* and where higher scores are reflective of greater perceived stress. The PSS has strong psychometric properties (Hewitt et al., 1992).

#### Parental anxiety: state-trait anxiety inventory (STAI)

Given associations with parental anxiety and children's pain, we assessed parent anxiety using the STAI, which is a widely used measure of state (situational) and trait (general) anxiety (Spielberger, 1983). The STAI is comprised of two 20-item measures assessed on a 4-point Likert-type scale ranging from 1 = *almost never* to 4 = *almost always* with higher scores reflecting higher anxiety. The STAI has excellent reliability and validity across multiple studies (Spielberger, 1989).

### Procedures

Children were recruited using lists of patients obtained from the cancer clinic from the hospitals at which recruitment occurred. Potentially eligible patients were contacted via telephone or in person at a scheduled appointment and screened for eligibility. Parents of eligible patients provided informed consent and children provide informed assent. All baseline measures were collected at consent using REDCap, an electronic data capture platform specifically designed, in part, for research data collection (Harris et al., 2009).

### Statistical analyses

Data screening procedures were performed using SPSS version 29 (IBM Corp., Armonk, NY, USA) and out-of-range values, skewness, kurtosis, means, and standard deviations (SD) were evaluated using descriptive statistics. The data were within the range of minimum and maximum values for each measure and the percentage of missing data overall was < 5%. Diagnoses were categorized based on the International Classification of Childhood Cancer (ICCC) as (1) leukemia, (2) lymphoma, (3) solid tumor, or (4) central nervous system tumor (SEER, https://seer.cancer.gov/iccc/). Demographic and descriptive variables were analyzed using frequencies. Multiple regression was used to examine associations between biopsychosocial variables, including diagnosis, time since diagnosis, child age, gender, race/ethnicity, annual income, physical functioning, pain-related self-efficacy, pain catastrophizing, fatigue, and anxiety and depressive symptoms, and parent anxiety symptoms and stress, and child pain intensity and pain interference ratings (Figure 1). Analyses were conducted in *Mplus* Version 8.10 using robust maximum likelihood estimation, which adjusts the standard errors and chi-square test statistics to account for multivariate kurtosis and data missingness.

## Results

Demographic and disease characteristics are presented in Table 1. Children were primarily male (53.2%) and White (41.4%), Latino (26.6%), or Asian (19.7%). The mean age of the sample was 12.3 years (SD = 2.86), and the average annual income was $87,758 (SD = $813.50; Median = $80,610). The majority of children who participated in the current study were receiving treatment for a leukemia (41.9%) or solid tumor (27.6%) diagnosis, and data were collected approximately 10 weeks following diagnosis (SD = 4.8). The mean pain intensity rating at time of data collection was 62.42 (SD = 22.4), indicating a moderate level of pain intensity on average.

**Table 1:** Demographic and disease characteristics.

**Variable**	***M* ±SD/*n* (%)**	**Range**
Child age (*n* = 197)	12.3 ± 2.86 years	8–18 years
**Child gender (*****n*** = **201)**		
Male	108 (53.2)	
Female	93 (46.8)	
**Child race/ethnicity (*****N*** = **203)**		
White	84 (41.4)	
Black/African American	20 (9.9)	
Latino	54 (26.6)	
Asian	40 (19.7)	
American Indian	5 (2.5)	
**Diagnosis category (*****N*** = **203)**		
Leukemia	85 (41.9)	
Lymphoma	32 (15.8)	
Solid Tumor	56 (27.6)	
Central nervous system tumor	30 (14.8)	
Time since diagnosis (weeks; *n* = 201)	9.9 ± 4.8	
Annual income (*n* = 161)	$87,758 ± 813.5	$350–$500,000
**Child self-report variables**		
Pain intensity (Visual analog scale; *N* = 203)	62.43 ± 22.4	30–100
Pain interference (*n* = 192)	31.2 ± 22.5	0–100
Physical functioning (*n* = 200)	60.77 ± 24.56	0–100
Pain self-efficacy (*n* =193)	19.1 ± 6.5	7–35
Pain catastrophizing (*n* = 193)	18.7 ± 11.1	0–52
Pain anxiety (*n* = 180)	31.1 ± 18.4	0–82
Fatigue (*n* = 200)	60.8 ± 24.6	0–100
Depressive symptoms (*n* = 199)	6.6 ± 3.6	0–17
Anxiety symptoms (*n* = 199)	21.7 ± 12.3	1–78
**Parent-proxy report variables**		
Physical functioning (*n* = 198)	47.91 ± 26.4	0–100
Pain self-efficacy (*n* = 194)	22.3 ± 6.4	7–35
Pain catastrophizing (*n* = 186)	21.1 ± 11.2	0–52
Fatigue (*n* = 198)	64.6 ± 18.0	4.2–100
Depressive symptoms (*n* = 200)	6.6 ± 4.0	0–22
Anxiety symptoms (*n* = 200)	19.7 ± 13.3	0–93
**Parent self-report variables**		
Anxiety symptoms (*n* = 175)	40.4 ± 9.03	24–66
Parental stress (*n* = 200)	24.9 ± 8.6	2–49

### Pain intensity

Multivariate regression analyses revealed that child age, gender, and fatigue, depressive symptoms, and pain catastrophizing were significantly associated with pain intensity. Specifically, older age (*Est*. = 1.95, *p* = 0.006), female gender (*Est*. = 7.32, *p* = 0.005), and elevated depressive symptoms (child self-report: *Est*. 1.87, *p* = 0.007; parent-proxy: *Est*. 2.16, *p* = 0.003), fatigue (child self-report: *Est*. = −0.40, *p* = 0.03; parent-proxy: *Est*. = −0.38, *p* = 0.02), and child self-reported pain catastrophizing (*Est*. 0.45, *p* = 0.036) were associated with higher pain intensity ratings. See Table 2 for results.

**Table 2:** Associations between biopsychosocial variables and pain intensity in children newly diagnosed with cancer.

**VAS**	**Estimate**	***p*-value**
**Child demographics**		
Age	1.945	0.006^**^
Gender	7.316	0.005^**^
Race/ethnicity	0.767	0.646
Annual income	0.009	0.690
Diagnosis	−1.867	0.318
Time since diagnosis	0.392	0.357
**Child self-report**		
Physical functioning	0.063	0.657
Pain self-efficacy	0.162	0.652
Pain catastrophizing	0.448	0.036^*^
Pain anxiety	0.161	0.291
Fatigue	−0.304	0.034^*^
Depressive symptoms	1.87	0.007^**^
Anxiety symptoms	0.010	0.962
**Parent-proxy report**		
Physical functioning	−0.039	0.744
Pain self-efficacy	−0.088	0.834
Pain catastrophizing	0.023	0.920
Fatigue	−0.380	0.020^*^
Depressive symptoms	2.164	0.003^**^
Anxiety symptoms	0.081	0.626
**Parent self-report**		
Anxiety symptoms	−0.016	0.956
Parental stress	0.271	0.389

^*^ = *p* < 0.05; ^**^ = *p* < 0.01.

### Pain interference

Annual income, physical functioning, fatigue, pain catastrophizing, and parent stress were significantly associated with pain interference. Specifically, lower annual income (*Est*. = −0.04, *p* = 0.001), decreased physical functioning (child self-report: *Est*. = −0.38, *p* < 0.001; parent-proxy: *Est*. = −0.28, *p* < 0.001), and greater fatigue (child self-report: *Est*. = −0.35, *p* = 0.029; parent-proxy: *Est*. = −0.30, *p* = 0.013), parent-proxy report of child pain catastrophizing (*Est*. = 0.35, *p* = 0.01), and parent self-report of stress (*Est*. = 0.62, *p* = 0.002) were significantly associated with child self-report of pain interference. See Table 3 for results.

**Table 3:** Associations between biopsychosocial variables and pain interference in children newly diagnosed with cancer.

**Pain-related disability**	**Estimate**	***p*-value**
**Child demographics**		
Age	0.479	0.255
Gender	−1.987	0.392
Race/ethnicity	−0.557	0.593
Annual income	−0.043	0.001^**^
Diagnosis	−0.617	0.574
Time since diagnosis	0.095	0.796
**Child self-report**		
Physical functioning	−0.377	< 0.001^**^
Pain self-efficacy	0.183	0.467
Pain catastrophizing	−0.169	0.334
Pain anxiety	0.168	0.085
Fatigue	−0.346	0.029
Depressive symptoms	−0.644	0.211
Anxiety symptoms	−0.129	0.314
**Parent-proxy report**		
Physical functioning	−0.276	< 0.001^**^
Pain self-efficacy	0.118	0.671
Pain catastrophizing	0.348	0.01^*^
Fatigue	−0.295	0.013^*^
Depressive symptoms	0.254	0.541
Anxiety symptoms	0.181	0.233
**Parent self-report**		
Anxiety symptoms	−0.341	0.101
Parental stress	0.619	0.002^**^

^*^= *p* < 0.05; ^**^ = *p* < 0.01.

## Discussion

The current study aimed to explore the biopsychosocial factors associated with pain experiences during the early phases of pediatric cancer treatment, with the goal of identifying children who may be at the highest risk for pain and pain-related disability to inform future intervention and prevention efforts. The results indicate that children who are older; female; from lower income families; reported heightened symptoms of depression, physical impairment, and fatigue, and pain catastrophizing; and who have parents who report elevated stress are at greatest risk for experiencing pain and pain-related disability in the early period after cancer diagnosis.

Findings of the current study underscore the importance of utilizing a comprehensive approach to pain assessment and management in pediatric oncology, highlighting the need for targeted interventions that consider the biological, psychological, and social contexts of patients. Older children may have a more developed understanding of their illness and treatment, which could lead to increased symptoms depression, ultimately heightening their perception of pain (Holley et al., 2017; King et al., 2011; Stanford et al., 2008). Similarly, the predominance of pain in female patients could suggest that sex and gender-specific factors, such as hormonal influences and differences in pain perception, play a significant role and thus warrant further investigation (King et al., 2011; Evans et al., 2010; Kløven et al., 2017). It may also be the case that consistent with societal influences and expectations, girls are more comfortable expressing pain and boys may be more hesitant to report pain and that both gender and developmental stage may influence pain reports (Boerner et al., 2014). Accordingly, both age (developmental status) and gender may be important contributors to pain expression in children with cancer. Additionally, addressing socioeconomic disparities is crucial to ensure equitable pain management and improve outcomes for all pediatric oncology patients. Lower annual income can negatively impact pain-related disability in children with cancer by limiting access to healthcare resources, increasing psychosocial stress, and exacerbating environmental stressors (Valvi et al., 2024; Dana Farber, 2022). Association between psychological symptoms, particularly depression, and pain experiences in this population aligns with existing literature that recognizes the interplay between emotional wellbeing and pain (Dudeney et al., 2024; Eccleston et al., 2004; Forgeron et al., 2013; Kashikar-Zuck et al., 2001, 2008). In this context, interventions designed to reduce depressive symptoms are crucial, as they target the emotional factors that contribute to the pain experience. Consequently, addressing these psychological symptoms could be pivotal in alleviating pain among children undergoing cancer treatment. This underscores the importance of integrating psychological assessments and interventions into standard care practices in pediatric oncology (Kazak et al., 2015), especially during the early stages of treatment.

Consistent with the pediatric chronic pain and oncology literature (Palermo et al., 2008; Duran et al., 2020; Ho et al., 2019; Konijnenberg et al., 2005; Madi and Clinton, 2018; Feinstein et al., 2017; Yu et al., 2024; Tutelman et al., 2022), the present study found associations between physical impairment pain catastrophizing, pain intensity, and pain interference. Children undergoing cancer treatment often experience significant disruptions to their daily activities and routines, which can exacerbate pain and increase the tendency to catastrophize pain, thereby impairing their ability to cope. Therefore, tailoring rehabilitation and physical therapy interventions to not only improve mobility but also address pain catastrophizing and enhance emotional resilience could significantly benefit children undergoing cancer treatment, aligning with a biopsychosocial treatment approach.

Fatigue was also found to play a significant role in the pain experience among children undergoing cancer treatment. Fatigue is common and distressing symptom of pediatric cancer treatment (Hooke and Linder, 2019) and it can exacerbate both the physical and emotional challenges associated with cancer, creating a vicious cycle that intensifies pain (Hockenberry et al., 2010). Therefore, recommended evidenced-based behavioral interventions for fatigue, such as exercise, physical activity promotion, and/or cognitive behavioral therapy (Patel et al., 2023), may also be effective at improving pain and pain-related disability in children receiving cancer treatment.

The influence of parental stress on pain cannot be overlooked. Findings of the current study indicate that caregivers' emotional states have a direct impact on child pain experiences, emphasizing the interconnectedness of family dynamics and child health (Bakula et al., 2020; Cowfer et al., 2023; Link and Fortier, 2016). Existing research suggests that children often absorb the emotional states of their caregivers, which can heighten their own anxiety and exacerbate pain (Palermo et al., 2014; Stassart et al., 2017; Tsao et al., 2006). This underscores the importance of utilizing a family-centered approach in pediatric cancer treatment. Providing parents with psychological support and resources may help alleviate their anxiety, which could, in turn, benefit their child's pain management (Bakula et al., 2020; Pai et al., 2007).

Overall, this study highlights the multifaceted nature of pain in the early phases of pediatric cancer treatment, advocating for a biopsychosocial assessment and treatment approach to cancer-related pain. Child age, gender, annual income, depressive symptoms, pain catastrophizing, physical impairment, and fatigue, and parental stress were found to be significantly associated with pain and pain-related disability among children during early phases of cancer treatment. Future research should focus on developing targeted interventions that address these identified biopsychosocial risk factors to ultimately improve pain management and quality of life for children undergoing cancer treatment. By recognizing and addressing the interplay between these factors, healthcare providers can deliver more effective, individualized interventions to improve pain in this vulnerable population.

Results of this study should be considered within the context of several limitations. First, to be eligible for the present study, children with cancer needed to score a 30 or greater on a VAS in the month prior to recruitment. Therefore, pain scores and contributing factors are likely to be more elevated among this sample of children, thus limiting the generalizability of current findings. Second, the cross-sectional design of the current study limits the ability to determine causal relationships between biopsychosocial factors and pain. While we conceptualized these variables primarily as risk factors for pain, it is also possible that the reverse direction of the effect is true—that higher pain levels contribute to increased psychological distress, greater pain-related disability, and heightened parental stress. Future longitudinal research is needed to clarify the directionality of these associations and better understand the dynamic interplay between pain and biopsychosocial factors over time. Third, self-report and parent proxy-report measures are subject to bias, and caregivers of children with cancer tend to report greater impairment compared to child-self report (Levi and Drotar, 1999; Pinquart and Kauser, 2018). Finally, the findings of the current study may be affected by confounding variables that were not controlled for in our analyses (i.e., intensity of treatment regimen, toxicity). As treatment intensity and toxicity have been shown to impact pain among survivors of pediatric cancer (Anderson and Woods, 2020), future research should consider controlling for these factors.

The findings of this study have significant implications for clinical practice, particularly for newly diagnosed pediatric cancer patients and their families. Routine assessment of pain and biopsychosocial risk factors should be conducted early and throughout pediatric cancer treatment. Integrating patient-reported outcome (PRO) measures into pediatric cancer care can enhance treatment decision-making and support a more personalized approach to care (Reeve et al., 2023; Horan et al., 2022; Lai et al., 2019). Given the multidimensional nature of pain, best practice consensus guidelines from the pain and oncology fields recommend, at a minimum, PROs assessing pain intensity and pain interference (McGrath et al., 2008; Palermo et al., 2024; Miale et al., 2019; Palermo et al., 2021). Additionally, PROs evaluating other critical domains, such as pain quality, location, frequency, health-related quality of life (HRQoL), and the impact of pain on emotional (e.g., depressive and anxiety symptoms) and physical functioning, as well as sleep quality, are strongly recommended to provide a comprehensive understanding of the pain (Palermo et al., 2024). Since depressive symptoms, physical impairments, fatigue, and pain catastrophizing were found to be significantly associated with pain intensity and interference in this study, routine assessment of these factors is essential to guide effective pain management strategies. Identifying children at higher risk for pain-related distress early in their cancer treatment can help clinicians implement targeted interventions, potentially reducing pain burden and improving overall quality of life for both patients and their families.

While physical activity and exercise have been shown to be beneficial for children undergoing cancer treatment (Baumann et al., 2013), early referral to physical therapy and rehabilitation services may help prevent loss of physical function, fatigue, and pain. However, it is important to recognize that newly diagnosed patients and their families may feel overwhelmed and may not have the time or resources to engage in extensive supportive care therapies initially. Brief, targeted interventions could be particularly beneficial at this stage. For example, short sessions of cognitive behavioral therapy (CBT) can help children cope with depression, pain catastrophizing, and fatigue. These brief therapies can be more manageable for families during the early phase of treatment and can still provide significant benefits. Additionally, given the influence of parental stress on children's pain experiences, family-centered treatment remains critical (Palermo, 2012). Providing support and resources for parents can reduce their stress levels, positively impacting their child's pain management. Brief CBT interventions for parents can help them understand the nature of their child's pain and the psychological factors involved, reducing their distress and improving their ability to support their child. For some families, the ability to engage with more comprehensive supportive care therapies may come later in the treatment process. Therefore, it is essential to offer flexible and adaptable support options that can be tailored to the family's readiness and capacity to engage.

## Data Availability

The raw data supporting the conclusions of this article will be made available by the authors, without undue reservation.

## References

[B1] Alberts N. M. Gagnon M. M. Stinson J. N. (2018). Chronic pain in survivors of childhood cancer: A developmental model of pain across the cancer trajectory. Pain 159, 1916–1927. 10.1097/j.pain.0000000000001261 29708940

[B2] Alberts N. M. Leisenring W. Whitton J. Stratton K. Jibb L. Flynn J.. (2024). Characterization of chronic pain, pain interference, and daily pain experiences in adult survivors of childhood cancer: a report from the childhood cancer survivor study. Pain 165, 2530–2543. 10.1097/j.pain.0000000000003284 38981063 PMC11474984

[B3] Anderson A. K. Woods S. (2020). Managing childhood cancer pain into survivorship: recognition and emerging principles. Curr. Opin. Support Palliat Care. 14:100. 10.1097/SPC.0000000000000492 32304399

[B4] Bakula D. M. Sharkey C. M. Perez M. N. Espeleta H. C. Gamwell K. L. Baudino M.. (2020). The relationship between parent distress and child quality of life in pediatric cancer: a meta-analysis. J. Pediatr. Nurs. 50, 14–19. 10.1016/j.pedn.2019.09.024 31670136

[B5] Baumann F. T. Bloch W. Beulertz J. (2013). Clinical exercise interventions in pediatric oncology: a systematic review. Pediatr. Res. 74, 366–374. 10.1038/pr.2013.123 23857296

[B6] Bevers K. Watts L. Kishino N. Gatchel R. (2016). The biopsychosocial model of the assessment, prevention, and treatment of chronic pain. US Neurol. 12:98. 10.17925/USN.2016.12.02.98

[B7] Boerner K. E. Birnie K. A. Caes L. Schinkel M. Chambers C. T. (2014). Sex differences in experimental pain among healthy children: a systematic review and meta-analysis. Pain 155, 983–993. 10.1016/j.pain.2014.01.031 24508752

[B8] Caes L. Vervoort T. Devos P. Verlooy J. Benoit Y. Goubert L.. (2014). Parental distress and catastrophic thoughts about child pain: implications for parental protective behavior in the context of child leukemia-related medical procedures. Clin. J. Pain. 30:787. 10.1097/AJP.0000000000000028 24042348

[B9] Chorpita B. F. Moffitt C. E. Gray J. (2005). Psychometric properties of the revised child anxiety and depression scale in a clinical sample. Behav. Res. Ther. 43, 309–322. 10.1016/j.brat.2004.02.004 15680928

[B10] Chorpita B. F. Yim L. Moffitt C. Umemoto L. A. Francis S. E. (2000). Assessment of symptoms of DSM-IV anxiety and depression in children: a revised child anxiety and depression scale. Behav. Res. Ther. 38, 835–855. 10.1016/S0005-7967(99)00130-8 10937431

[B11] Cohen S. Kamarck T. Mermelstein R. A. (1983). Global measure of perceived stress. J. Health Soc. Behav. 24, 385–396. 10.2307/2136404 6668417

[B12] Collins J. J. Byrnes M. E. Dunkel I. J. Lapin J. Nadel T. Thaler H. T.. (2000). The measurement of symptoms in children with cancer. J. Pain Symptom. Manage. 19, 363–377. 10.1016/S0885-3924(00)00127-5 10869877

[B13] Coluzzi F. Rocco M. Green Gladden R. Persiani P. Thur L. A. Milano F.. (2020). Pain management in childhood leukemia: diagnosis and available analgesic treatments. Cancers 12:3671. 10.3390/cancers12123671 33297484 PMC7762342

[B14] Cowfer B. A. Dietrich M. S. Akard T. F. Gilmer M. J. (2023). Relationships between parental anxiety and child quality of life in advanced childhood cancer. J. Pediatr. Hematol. Nurs. 40, 209–216. 10.1177/27527530221147876 37032466 PMC12500218

[B15] Crombez Bijttebier P. Eccleston C. Mascagni T. Mertens G. Goubert L. Verstraeten K. G.. (2003). The child version of the pain catastrophizing scale (PCS-C): a preliminary validation. Pain 104, 639–649. 10.1016/S0304-3959(03)00121-0 12927636

[B16] Dana Farber (2022). Addressing the Impact of Poverty on Childhood Cancer. Dana-Farber Cancer Institute.

[B17] de Oliveira A. M. B. da Teixeira D. S. C. dos Menezes F. S. Marques A. P. de Duarte Y. A. O. Casarotto R. A. (2023). Socioeconomic and sex inequalities in chronic pain: a population-based cross-sectional study. PLoS ONE. 18:e0285975. 10.1371/journal.pone.0285975 37228121 PMC10212187

[B18] Diller L. Chow E. J. Gurney J. G. Hudson M. M. Kadan-Lottick N. S. Kawashima T. I.. (2009). Chronic disease in the childhood cancer survivor study cohort: a review of published findings. J. Clin. Oncol. 27, 2339–2355. 10.1200/JCO.2008.21.1953 19364955 PMC2677922

[B19] Dudeney J. Aaron R. V. Hathway T. Bhattiprolu K. Bisby M. A. McGill L. S.. (2024). Anxiety and depression in youth with chronic pain: a systematic review and meta-analysis. JAMA Pediatr. 178, 1114–1123. 10.1001/jamapediatrics.2024.3039 39250143 PMC11385330

[B20] Dupuis L. L. Lu X. Mitchell H. R. Sung L. Devidas M. Mattano Jr L. A.. (2016). Anxiety, pain, and nausea during the treatment of standard-risk childhood acute lymphoblastic leukemia: a prospective, longitudinal study from the Children's Oncology Group. Cancer 122, 1116–1125. 10.1002/cncr.29876 26773735 PMC5138861

[B21] Duran J. Bravo L. Torres V. Craig A. Heidari J. Adlard K.. (2020). Quality of life and pain experienced by children and adolescents with cancer at home following discharge from the hospital. J. Pediatr. Hematol. Oncol. 42:46. 10.1097/MPH.0000000000001605 31725538 PMC6920561

[B22] Eccleston C. Crombez G. Scotford A. Clinch J. Connell H. (2004). Adolescent chronic pain: patterns and predictors of emotional distress in adolescents with chronic pain and their parents. Pain 108, 221–229. 10.1016/j.pain.2003.11.008 15030941

[B23] Elliott S. C. Miser A. W. Dose A. M. Betcher D. L. O'Fallon J. R. Ducos R. S.. (1991). Epidemiologic features of pain in pediatric cancer patients: a co-operative community-based study. North central cancer treatment group and mayo clinic. Clin. J. Pain. 7, 263–268. 10.1097/00002508-199112000-00003 1809439

[B24] Evans S. Taub R. Tsao J. C. Meldrum M. Zeltzer L. K. (2010). Sociodemographic factors in a pediatric chronic pain clinic: the roles of age, sex and minority status in pain and health characteristics. J. Pain Manag. 3, 273–281. 21686073 PMC3113686

[B25] Feinstein A. B. Sturgeon J. A. Darnall B. D. Dunn A. L. Rico T. Kao M. C.. (2017). The effect of pain catastrophizing on outcomes: a developmental perspective across children, adolescents, and young adults with chronic pain. J. Pain. 18, 144–154. 10.1016/j.jpain.2016.10.009 27825857 PMC5291801

[B26] Forgeron P. Evans J. McGrath P. Stevens B. J. Finley G. A. (2013). Living with difference: exploring the social self of adolescents with chronic pain. Pain Res. Manag. 18, e115–e123. 10.1155/2013/120632 24308027 PMC3917802

[B27] Forgeron P. A. Finley G. A. Arnaout M. (2006). Pediatric pain prevalence and parents' attitudes at a cancer hospital in Jordan. J. Pain Symptom. Manage. 31, 440–448. 10.1016/j.jpainsymman.2005.09.003 16716874

[B28] Fuller C. Huang H. Thienprayoon R. (2022). Managing pain and discomfort in children with cancer. Curr. Oncol. Rep. 24, 961–973. 10.1007/s11912-022-01277-1 35353347

[B29] Gatchel R. J. McGeary D. D. McGeary C. A. Lippe B. (2014). Interdisciplinary chronic pain management: past, present, and future. Am. Psychol. 69:119. 10.1037/a0035514 24547798

[B30] Gatchel R. J. Peng Y. B. Peters M. L. Fuchs P. N. Turk D. C. (2007). The biopsychosocial approach to chronic pain: scientific advances and future directions. Psychol. Bull. 133:581. 10.1037/0033-2909.133.4.581 17592957

[B31] Harris P. A. Taylor R. Thielke R. Payne J. Gonzalez N. Conde J. G.. (2009). Research electronic data capture (REDCap)–a metadata-driven methodology and workflow process for providing translational research informatics support. J. Biomed. Inform. 42, 377–381. 10.1016/j.jbi.2008.08.010 18929686 PMC2700030

[B32] Hedstrom M. Haglund K. Skolin I. von Essen L. (2003). Distressing events for children and adolescents with cancer: child, parent, and nurse perceptions. J. Pediatr. Oncol. Nurs. 20, 120–132. 10.1053/jpon.2003.76 12776260

[B33] Hewitt P. L. Flett G. L. Mosher S. W. (1992). The perceived stress scale: factor structure and relation to depression symptoms in a psychiatric sample. J. Psychopathol. Behav. Assess. 14, 247–257. 10.1007/BF00962631

[B34] Ho K. Y. Li W. H. C. Lam K. W. K. Wei X. Chiu S. Y. Chan C. F. G.. (2019). Relationships among fatigue, physical activity, depressive symptoms, and quality of life in Chinese children and adolescents surviving cancer. Eur. J. Oncol. Nurs. 38, 21–27. 10.1016/j.ejon.2018.11.007 30717932

[B35] Hockenberry M. J. Hooke M. C. Gregurich M. McCarthy K. Sambuco G. Krull K.. (2010). Symptom clusters in children and adolescents receiving cisplatin, doxorubicin, or ifosfamide. Oncol. Nurs. Forum. 37, E16–E27. 10.1188/10.ONF.E16-E27 20044328

[B36] Holley A. L. Wilson A. C. Palermo T. M. (2017). Predictors of the transition from acute to persistent musculoskeletal pain in children and adolescents: a prospective study. Pain 158, 794–801. 10.1097/j.pain.0000000000000817 28151835 PMC5393939

[B37] Hooke M. C. Linder L. A. (2019). Symptoms in children receiving treatment for cancer-part I: fatigue, sleep disturbance, and nausea/vomiting. J. Pediatr. Oncol. Nurs. 36, 244–261. 10.1177/1043454219849576 31307321 PMC7197223

[B38] Horan M. R. Sim J. Krull K. R. Baker J. N. Huang I. C. (2022). A review of patient-reported outcome measures in childhood cancer. Children 9:1497. 10.3390/children9101497 36291433 PMC9601091

[B39] Huang I. C. Brinkman T. M. Kenzik K. Gurney J. G. Ness K. K. Lanctot J.. (2013). Association between the prevalence of symptoms and health-related quality of life in adult survivors of childhood cancer: a report from the st jude lifetime cohort study. J. Clin. Oncol. 31, 4242–4251. 10.1200/JCO.2012.47.8867 24127449 PMC3821013

[B40] Huguet A. Tougas M. E. Hayden J. McGrath P. J. Chambers C. T. Stinson J. N.. (2016). Systematic review of childhood and adolescent risk and prognostic factors for recurrent headaches. J. Pain. 17, 855–873.e8. 10.1016/j.jpain.2016.03.010 27102894

[B41] Jibb L. A. Ameringer S. Macpherson C. F. Sivaratnam S. (2022). The symptom experience in pediatric cancer: current conceptualizations and future directions. Curr. Oncol. Rep. 24, 443–450. 10.1007/s11912-022-01222-2 35150393

[B42] Kashikar-Zuck S. Goldschneider K. R. Powers S. W. Vaught M. H. Hershey A. D. (2001). Depression and functional disability in chronic pediatric pain. Clin. J. Pain. 17, 341–349. 10.1097/00002508-200112000-00009 11783815

[B43] Kashikar-Zuck S. Parkins I. S. Graham T. B. Lynch A. M. Passo M. Johnston M.. (2008). Anxiety, mood, and behavioral disorders among pediatric patients with juvenile fibromyalgia syndrome. Clin. J. Pain. 24, 620–626. 10.1097/AJP.0b013e31816d7d23 18716501 PMC3711138

[B44] Kazak A. E. Schneider S. Didonato S. Pai A. L. H. (2015). Family psychosocial risk screening guided by the pediatric psychosocial preventative health model (PPPHM) using the psychosocial assessment tool (PAT). Acta. Oncol. 54, 574–580. 10.3109/0284186X.2014.995774 25752970

[B45] King S. Chambers C. T. Huguet A. MacNevin R. C. McGrath P. J. Parker L.. (2011). The epidemiology of chronic pain in children and adolescents revisited: a systematic review. Pain 152, 2729–2738. 10.1016/j.pain.2011.07.016 22078064

[B46] Klages K. L. Gibson C. A. Barnett K. A. Schwartz L. E. Hicks C. A. Norris R. E.. (2025). Systematic review of pain assessment measures used in pediatric acute lymphoblastic leukemia. Psychooncology 34:e70063. 10.1002/pon.70063 39746810

[B47] Kløven B. Hoftun G. B. Romundstad P. R. Rygg M. (2017). Relationship between pubertal timing and chronic nonspecific pain in adolescent girls: the young-HUNT3 study (2006–2008). Pain 158:1554. 10.1097/j.pain.0000000000000950 28520646

[B48] Konijnenberg A. Y. Uiterwaal C. S. Kimpen J. L. van der Hoeven J. Buitelaar J. K. Graeff-Meeder E. R.. (2005). Children with unexplained chronic pain: substantial impairment in everyday life. Arch. Dis. Child. 90, 680–686. 10.1136/adc.2004.056820 15899922 PMC1720481

[B49] Kovačević I. Pavić J. Filipović B. Ozimec Vulinec Š. Ilić B. Petek D.. (2024). Integrated approach to chronic pain-the role of psychosocial factors and multidisciplinary treatment: a narrative review. Int. J. Environ. Res. Public Health. 21:1135. 10.3390/ijerph21091135 39338018 PMC11431289

[B50] Kremer R. Granot M. Yarnitsky D. Crispel Y. Fadel S. Anson Best L.. (2013). The role of pain catastrophizing in the prediction of acute and chronic postoperative pain. Open Pain J. 6, 176–182. 10.2174/1876386301306010176

[B51] Lai J. S. Kupst M. J. Beaumont J. L. Manley P. E. Chang J. H. C. Hartsell W. F.. (2019). Using the patient-reported outcomes measurement information system (PROMIS) to measure symptom burden reported by patients with brain tumors. Pediatr. Blood Cancer. 66:e27526. 10.1002/pbc.27526 30426667 PMC6344265

[B52] Levi R. B. Drotar D. (1999). Health-related quality of life in childhood cancer: discrepancy in parent-child reports. Int. J. Cancer Suppl. J. Int. Cancer Suppl. 12, 58–64. 10.1002/(SICI)1097-0215(1999)83:12+<58::AID-IJC11>3.0.CO;2-A 10679872

[B53] Levine D. R. Mandrell B. N. Sykes A. Pritchard M. Gibson D. Symons H. J.. (2017). Patients' and parents' needs, attitudes, and perceptions about early palliative care integration in pediatric oncology. JAMA Oncol. 3, 1214–1220. 10.1001/jamaoncol.2017.0368 28278329 PMC5824292

[B54] Lin W. Y. Hsieh J. C. Lu C. C. Ono Y. (2022). Altered metabolic connectivity between the amygdala and default mode network is related to pain perception in patients with cancer. Sci. Rep. 12:14105. 10.1038/s41598-022-18430-2 35982228 PMC9388574

[B55] Link C. J. Fortier M. A. (2016). The relationship between parent trait anxiety and parent-reported pain, solicitous behaviors, and quality of life impairment in children with cancer. J. Pediatr. Hematol. Oncol. 38, 58–62. 10.1097/MPH.0000000000000376 26056789

[B56] Liossi C. Howard R. F. (2016). Pediatric chronic pain: biopsychosocial assessment and formulation. Pediatrics 138:e20160331. 10.1542/peds.2016-0331 27940762

[B57] Lu Q. Krull K. R. Leisenring W. Owen J. E. Kawashima T. Tsao J. C.. (2011). Pain in long-term adult survivors of childhood cancers and their siblings: a report from the childhood cancer survivor study. Pain 152, 2616–2624. 10.1016/j.pain.2011.08.006 21907493 PMC3304496

[B58] Madi D. Clinton M. (2018). Pain and its impact on the functional ability in children treated at the children's cancer center of Lebanon. J. Pediatr. Nurs. 39, e11–e20. 10.1016/j.pedn.2017.12.004 29338904

[B59] McGrath P. J. Walco G. A. Turk D. C. Dworkin R. H. Brown M. T. Davidson K.. (2008). Core outcome domains and measures for pediatric acute and chronic/recurrent pain clinical trials: PedIMMPACT recommendations. J. Pain. 9, 771–783. 10.1016/j.jpain.2008.04.007 18562251

[B60] Meints S. M. Edwards R. R. (2018). Evaluating psychosocial contributions to chronic pain outcomes. Prog. Neuropsychopharmacol. Biol. Psychiatry. 87, 168–182. 10.1016/j.pnpbp.2018.01.017 29408484 PMC6067990

[B61] Miale S. Harrington S. Brown K. Braswell A. Cannoy J. Krisch N.. (2019). Academy of oncologic physical therapy EDGE task force on cancer: a systematic review of outcome measures for pain in children. Rehabil. Oncol. 37:47. 10.1097/01.REO.0000000000000165

[B62] Mills S. E. E. Nicolson K. P. Smith B. H. (2019). Chronic pain: a review of its epidemiology and associated factors in population-based studies. Br. J. Anaesth. 123, e273–283. 10.1016/j.bja.2019.03.023 31079836 PMC6676152

[B63] Miser A. W. McCalla J. Dothage J. A. Wesley M. Miser J. S. (1987). Pain as a presenting symptom in children and young adults with newly diagnosed malignancy. Pain 29, 85–90. 10.1016/0304-3959(87)90181-3 3588003

[B64] Moody K. Meyer M. Mancuso C. A. Charlson M. Robbins L. (2006). Exploring concerns of children with cancer. Support Care Cancer. 14, 960–966. 10.1007/s00520-006-0024-y 16639553

[B65] Page G. G. (2013). “The immune-suppressive effects of pain,” in Madame Curie Bioscience Database (Austin, TX: Landes Bioscience). Available online at: https://www.ncbi.nlm.nih.gov/books/NBK6140/

[B66] Pai A. L. H. Greenley R. N. Lewandowski A. Drotar D. Youngstrom E. Peterson C. C. A.. (2007). meta-analytic review of the influence of pediatric cancer on parent and family functioning. J. Fam. Psychol. 21, 407–415. 10.1037/0893-3200.21.3.407 17874926

[B67] Palermo T. M. (2012). Cognitive-Behavioral Therapy for Chronic Pain in Children and Adolescents. New York, NY: Oxford University Press. 10.1093/med:psych/9780199763979.001.0001

[B68] Palermo T. M. Lewandowski A. S. Long A. C. Burant C. J. (2008). Validation of a self-report questionnaire version of the child activity limitations interview (CALI): the CALI-21. Pain 139, 644–652. 10.1016/j.pain.2008.06.022 18692316 PMC3166250

[B69] Palermo T. M. Li R. Birnie K. A. Crombez G. Eccleston C. Kashikar-Zuck S.. (2024). Updated recommendations on measures for clinical trials in pediatric chronic pain: a multiphase approach from the core outcomes in pediatric persistent pain (Core-OPPP) Workgroup. Pain 165:1086. 10.1097/j.pain.0000000000003105 38112633 PMC11017748

[B70] Palermo T. M. Valrie C. R. Karlson C. W. (2014). Family and parent influences on pediatric chronic pain. Am. Psychol. 69, 142–152. 10.1037/a0035216 24547800 PMC4056332

[B71] Palermo T. M. Walco G. A. Paladhi U. R. Birnie K. A. Crombez G. de la Vega R. (2021). Core outcome set for pediatric chronic pain clinical trials: results from a Delphi poll and consensus meeting. Pain 162, 2539–2547. 10.1097/j.pain.0000000000002241 33625074 PMC8442740

[B72] Patel P. Robinson P. D. van der Torre P. Tomlinson D. Seelisch J. Oberoi S.. (2023). Guideline for the management of fatigue in children and adolescents with cancer or pediatric hematopoietic cell transplant recipients: 2023 update. eClinicalMedicine. 63:102147. 10.1016/j.eclinm.2023.102147 37609066 PMC10440444

[B73] Pinquart M. Kauser R. (2018). Do the associations of parenting styles with behavior problems and academic achievement vary by culture? Results from a meta-analysis. Cultur. Divers. Ethnic. Minor. Psychol. 24, 75–100. 10.1037/cdp0000149 28394165

[B74] Pöder U. Ljungman G. von Essen L. (2010). Parents' perceptions of their children's cancer-related symptoms during treatment: a prospective, longitudinal study. J. Pain Symptom. Manage. 40, 661–670. 10.1016/j.jpainsymman.2010.02.012 20678894

[B75] Reeve B. B. Greenzang K. A. Sung L. (2023). Patient-reported outcomes in pediatric patients with cancer. Am. Soc. Clin. Oncol. Educ. Book. 43:e390272. 10.1200/EDBK_390272 37172266 PMC11299117

[B76] Ribeiro I. L. A. Limeira R. R. T. de Dias Castro R. Ferreti Bonan P. R. Valença A. M. G. (2017). Oral mucositis in pediatric patients in treatment for acute lymphoblastic leukemia. Int. J. Environ. Res. Public Health. 14:1468. 10.3390/ijerph14121468 29182564 PMC5750887

[B77] Ruccione K. Lu Y. Meeske K. (2013). Adolescents' psychosocial health-related quality of life within 6 months after cancer treatment completion. Cancer Nurs. 36, E61–72. 10.1097/NCC.0b013e3182902119 23632469

[B78] Savedra M. Tesler M. Holzemer W. Ward J. (1989). Adolescent Pediatric Pain Tool (APPT): Preliminary User's Manual. San Francisco: University of California.

[B79] Schmitz A. K. Vierhaus M. Lohaus A. (2013). Pain tolerance in children and adolescents: Sex differences and psychosocial influences on pain threshold and endurance. Eur. J. Pain. 17, 124–131. 10.1002/j.1532-2149.2012.00169.x 22715044

[B80] Siegel R. L. Miller K. D. Fuchs H. E. Jemal A. (2021). Cancer statistics, 2021. CA Cancer J. Clin. 71, 7–33. 10.3322/caac.21654 33433946

[B81] Silva Santos Ribeiro P. Willemen H. L. D. M. Eijkelkamp N. (2022). Mitochondria and sensory processing in inflammatory and neuropathic pain. Front. Pain. Res. 3:1013577. 10.3389/fpain.2022.1013577 36324872 PMC9619239

[B82] Simons L. E. Elman I. Borsook D. (2014). Psychological processing in chronic pain: a neural systems approach. Neurosci. Biobehav. Rev. 39, 61–78. 10.1016/j.neubiorev.2013.12.006 24374383 PMC3944001

[B83] Spielberger C. (1989). State-Trait Anxiety Inventory: A Comprehensive Bibliography. Anonymous, editor. Palo Alto, CA: Mind Garden.

[B84] Spielberger C. D. (1983). Manual for the State-Trait Anxiety Inventory (STAI : Form Y). Palo Alto, CA: Consulting Psychologists Press, 4–26. 10.1037/t06496-000

[B85] Stanford E. A. Chambers C. T. Biesanz J. C. Chen E. (2008). The frequency, trajectories and predictors of adolescent recurrent pain: a population-based approach. Pain 138, 11–21. 10.1016/j.pain.2007.10.032 18093737

[B86] Stassart C. Dardenne B. Etienne A. M. (2017). The role of parental anxiety sensitivity and learning experiences in children's anxiety sensitivity. Br. J. Dev. Psychol. 35, 359–375. 10.1111/bjdp.12172 28120529

[B87] Tsao J. C. I. Lu Q. Myers C. D. Kim S. C. Turk N. Zeltzer L. K.. (2006). Parent and child anxiety sensitivity: relationship to children's experimental pain responsivity. J. Pain. 7, 319–326. 10.1016/j.jpain.2005.12.004 16632321 PMC1540407

[B88] Tutelman P. R. Chambers C. T. Noel M. Heathcote L. C. Fernandez C. V. Flanders A.. (2022). Pain and fear of cancer recurrence in survivors of childhood cancer. Clin. J. Pain. 38, 484–491. 10.1097/AJP.0000000000001049 35686578

[B89] Tutelman P. R. Chambers C. T. Stinson J. N. Parker J. A. Fernandez C. V. Witteman H. O.. (2018). Pain in children with cancer: prevalence, characteristics, and parent management. Clin. J. Pain. 34, 198–206. 10.1097/AJP.0000000000000531 28678061

[B90] Twycross A. Parker R. Williams A. Gibson F. (2015). Cancer-related pain and pain management: sources, prevalence, and the experiences of children and parents. J. Pediatr. Oncol. Nurs. 32, 369–384. 10.1177/1043454214563751 25736032

[B91] Uhl K. Burns M. Hale A. Coakley R. (2020). The critical role of parents in pediatric cancer-related pain management: a review and call to action. Curr. Oncol. Rep. 22:37. 10.1007/s11912-020-0899-7 32172378

[B92] Valvi N. Tamargo J. A. Braithwaite D. Fillingim R. B. Karanth S. D. (2024). Household income is associated with chronic pain and high-impact chronic pain among cancer survivors: a cross-sectional study using NHIS data. Cancers 16:2847. 10.3390/cancers16162847 39199618 PMC11353052

[B93] Varni J. W. Burwinkle T. M. Katz E. R. Meeske K. Dickinson P. (2002). The PedsQL in pediatric cancer: reliability and validity of the pediatric quality of life inventory generic core scales, multidimensional fatigue scale, and cancer module. Cancer 94, 2090–2106. 10.1002/cncr.10428 11932914

[B94] Varni J. W. Limbers C. A. (2009). The PedsQLTM 4.0 generic core scales young adult version feasibility, reliability and validity in a university student population. J. Health Psychol. 14, 611–622. 10.1177/1359105309103580 19383661

[B95] Vierhaus M. Lohaus A. Schmitz A. K. (2011). Sex, gender, coping, and self-efficacy: Mediation of sex differences in pain perception in children and adolescents. Eur. J. Pain. 15, 621.e1–621.e8. 10.1016/j.ejpain.2010.11.003 21147542

[B96] Yu Q. Fang F. Chen L. Wang Q. The D. a. i. W. (2024). Relationship of pain catastrophizing in principal caregivers of postoperative children with malignant bone tumors and children's kinesiophobia and pain perception: a cross-sectional survey. Int. J. Orthop. Trauma. Nurs. 55:101137. 10.1016/j.ijotn.2024.101137 39307042

[B97] Zernikow B. Meyerhoff U. Michel E. Wiesel T. Hasan C. Janssen G.. (2005). Pain in pediatric oncology–children's and parents' perspectives. Eur. J. Pain. 9, 395–406. 10.1016/j.ejpain.2004.09.008 15979020

